# Pot study using *Chlorophytum comosum* plants to biomonitor PAH levels in domestic kitchens

**DOI:** 10.1007/s11356-023-25469-9

**Published:** 2023-02-23

**Authors:** Katalin Hubai, Nora Kováts, Bettina Eck-Varanka, Gábor Teke

**Affiliations:** 1 University of Pannonia, Centre for Natural Sciences, Egyetem Str. 10, Veszprém, 8200 Hungary; 2 ELGOSCAR-2000 Environmental Technology and Water Management Ltd., Balatonfűzfő, 8184 Hungary

**Keywords:** Indoor air quality, Cooking, polycyclic aromatic hydrocarbons, biomonitoring, *Chlorophytum comosum*

## Abstract

In indoor environments, cooking is a major contributor to indoor air pollution releasing potentially harmful toxic compounds such as polycyclic aromatic hydrocarbons. In our study, *Chlorophytum comosum* ‘Variegata’ plants were applied to monitor PAH emission rates and patterns in previously selected rural Hungarian kitchens. Concentration and profile of accumulated PAHs could be well explained by cooking methods and materials used in each kitchen. Accumulation of 6-ring PAHs was characteristic in the only kitchen which frequently used deep frying. It also should be emphasized that applicability of *C. comosum* as indoor biomonitor was assessed. The plant has proven a good monitor organism as it accumulated both LMW and HMW PAHs.

## Introduction

In Europe, people spend approximately 90% of their time indoors (González-Martín et al. 2021), which has raised the necessity to investigate indoor air pollution levels and their potential health impacts. Most studies agree that cooking is a major contributor to indoor air pollution (Zhai and Albritton 2020). During cooking, particulate matter (PM) can arise from the food source, fuel used and from the coating layers of heated cookware (Cho et al. 2019). Considering the number of emitted particles, the dominant fractions are ultrafine particles (UFPs) and accumulation mode particles (AMPs) (Li et al. 2017a). In the study of Wallace and Ott (2011), cooking was one of the greatest indoor sources of ultrafine particles (UFP). The smaller the more dangerous: smaller particles bind relatively more potentially toxic compounds due to the relatively bigger surface. Also, submicron particles can penetrate the respiratory system deep causing serious health risks (Zhang et al. 2017).

Deep-frying is one of the most prevailing cooking methods (Yao et al. 2015, Liu et al. 2019, Touffet et al. 2020). Most studies agree that frying produces higher particle emissions compared to steaming and boiling (reviewed by Torkmahalleh et al. 2017). It is usually conducted at high temperatures (150 to 190 °C) which favors the emission of polycyclic aromatic hydrocarbons (PAHs). In many households, the same batch of oil is used on several occasions which further increase the risk of PAH emissions (Ng et al. 2014, Hao et al. 2016, Juániz et al. 2016). Iwegbue et al. (2020) measured PAH content in different vegetable oils before use and after three successive cycles of frying, reporting significant increases. Raw material can also influence emission rates, for example preparing fatty meats generate higher emissions than vegetables (reviewed by Li 2021).

Several studies have reported the dominance of naphthalene (Nap) in different kitchens (e.g., Zhu and Wang 2003, Wang et al. 2015). Nap mainly occurs in the gaseous phase; its relative share might reach as much as 89% of the total gaseous PAHs (Chen et al. 2007). It was for example detected in the breathing area of kitchen workers (Sjaastad and Svendsen, 2009). Huang et al. (2021) compared volatile organic compounds emission in frying, steaming, and grilling commercial kitchens and found that the three cooking styles had similar production of Nap. Its metabolite 2-hydroxynaphthalene was also detected in the urine sample of kitchen workers, indicating oxidative stress. It is a two-ring, low molecular weight PAH which is considered an ubiquitous environmental pollutant (Preuss et al. 2003). Based on the degree of evidence for the carcinogenicity, the International Agency for Research on Cancer (IARC) identified naphthalene as “possibly carcinogenic to humans” (group 2B) (IARC 2010).

While active sampling has been widely applied to characterise the risk of PM and especially PAH emissions during cooking, biomonitoring studies have been mostly restricted to human samples (Li et al. 2011, Oliveira et al. 2017, Murawski et al. 2020). Neupane et al. (2019), e.g., used hair samples of cooks to estimate health risk of chronic exposure in restaurants. Considering non-human samples, spider webs were used in the studies of Rutkowski et al. (2019) and Rybak et al. (2019) to monitor indoor PAH levels. Spider webs can be collected *in situ* (van Laaten et al. 2020), while other traditional biomonitors such as lichens need to be transplanted. Transplanted *Pseudovernia furfuracea* plants were used in the study of Protano et al. (2017) in indoor school environments. Lichen samples accumulated both heavy metals and PAHs. Transplanted lichens, however, might face a certain level of physiological stress caused by specific conditions such as temperature and humidity (Canha et al. 2012). Paoli et al. (2019) placed *Evernia prunastri* in school environments and found that in two months exposure physiological impairment of the samples could be avoided, and this time was sufficient for the test plants to bioaccumulate heavy metals.

Biomonitors are considered a cheap and viable solution for monitoring and show adequate sensitivity even in case of relatively low contamination level (Baldantoni and Alfani 2016). Results will provide a time-averaged data series (Zhao et al. 2018). It might be especially important in kitchen environments where emission intensity can show high temporal variations: for example, fine particulate matter (PM2.5) concentrations increased by a factor of as much as 85 during cooking compared to non-cooking time in the study of See and Balasubramanian (2006).

In our study, *Chlorophytum comosum* ‘Variegata’ (Thunb.) Jacques (Family Asparagaceae, spider plant, syn. spider ivy, ribbon plant, or hen and chickens) plants were applied to monitor PAH emission rates and patterns in previously selected kitchens. The species is native to tropical and southern Africa but has become a widely cultivated ornamental plant in Europe.

*C. comosum* has proven a good candidate for phytoremediation of pollutants such as benzene (Sriprapat and Strand 2016), ethylbenzene (Sriprapat et al. 2014), toluene (Treesubsuntorn and Thiravetyan 2018), and particulate matter (Gawrońska and Bakera 2015) from indoor air. *C. comosum* was reported to have one of the highest VOC removal efficiency in comparison to other plant species tested (Treesubsuntorn et al. 2020). The species could take up and break down formaldehyde in the leaves (Su and Liang 2015).

Morphological characteristics make the plant a promising accumulator. Good bioaccumulator capacity is generally attributed to the following morphological parameters: leaf size/shape (plants with high surface-to-volume ratio; roughness and/or hairiness of the leaves) (Franzaring and van der Eerden 2000); presence of epicuticular wax (Li et al. 2017b). The plant has long narrow leaves (length 20–45 cm and width 6–25 millimetres). Leaves also contain a well-developed wax layer (Fermo et al. 2021). *C. comosum* is also a good choice for pot studies as the plant reproduces vegetatively and genetically uniform test material can be produced. The plant tolerates semi-shade, making it useable in indoor environments.

In the study of Fermo et al. (2021), *C. comosum* was applied to biomonitor heavy metal levels in different sites of Milan (Italy). The species has adapted to the climatic conditions of Milan, therefore outdoor air quality could be monitored with special regard of heavy metal levels such as Zn, Mn, Cd, Cr, Co, Ni, and Pb.

While a wide range of studies have addressed the magnitude and impact of emissions generated by Asian cooking (Feng et al. 2021; Lin et al. 2019), much less data are available for European domestic kitchens (e.g., Alves et al. 2021). According to pre-COVID studies, the role of eating out in Hungary has been low compared to international habits (Lehota et al. 2015) which might emphasize the importance to study human exposure to cooking-generated pollution. Hungarian cooking can also be regarded as specific in comparison to Western European style taking into consideration the relatively high share of lard (Grasgruber et al. 2018).

The main goal of our study was that in addition to gaining a comprehensive picture about PAH exposure in typical rural Hungarian kitchens, we wanted to examine and establish the applicability of *C. comosum* for indoor bioaccumulation studies.

## Material and methods

### Pot study

*C. comosum* ‘Variegatum’ plant was purchased from a local retainer. Plantlets of similar size were separated. They were acclimatized in uncontaminated commercial soil (pH: 6.8 ± 0.5; N (m/m%): min 0.3; P_2_O_5_ (m/m%): min 0.1; and K_2_O (m/m%): min 0.3) and placed in a greenhouse for four weeks before the study. In each kitchen, 4 plants were used.

Exposure started 1 June and ended 31 July. This period was chosen to avoid potential cross-pollution from heating. Normally heating season ends in April in Hungary but May was exceptionally cold in 2021, therefore several households continued heating in May. Another reason was for selection that during summer holidays children mostly lunch at home, which increases cooking frequency.

After the first month, 1-month-old leaves while after the second month, 2-month-old leaves were selected and cut with pre-washed scissors (using ultrapure water and ethanol). Leaves were immediately taken to the laboratory, washed, freeze dried, ground, homogenized (Wang et al. 2021), and kept in the freezer (− 20 °C) until analysis.

### Household selection

Households with approximately the similar size were selected (2 adults + 2 children) (Table 1). Another important criterion was the distance from main roads: all households are situated in small villages, not affected by heavy traffic. This criterion was especially important as some indoor biomonitoring studies reported that in case of traffic-impacted sites such as schools, infiltration of outdoor air pollutants could be experienced (Paoli et al. 2019, Protano et al. 2017).

**Table 1:** Key data for the surveyed households

	HH1	HH2	HH3	HH4
Number of inhabitants	4	4	4	4
Cooking frequency per day	1, very seldom 2 (less than 5%)	Usually 2	Usually 2	1, very seldom 2 (less than 5%)
Energy source	Electric stove/oven	Gas stove Electric oven	Electric stove/oven	Electric stove/oven
Material used	Lard approximately 40% Vegetable (sunflower) oil approximately 55% Olive oil approximately 5%	Lard approximately 95% Butter approximately 5%	Lard 1% vegetable (sunflower) oil approximately 89%, coconut approximately 10%	Vegetable (sunflower) oil approximately 100%
Cooking method	Boiling 35% Deep-frying 30% Pan-frying 15% Oven 20%	Boiling 40% Deep-frying 0% Pan-frying 40% Oven 20%	Boiling 30% Deep frying 55% Pan frying 5% Oven 10%	Boiling 50% Deep-frying 15% Pan-frying 5% Oven 30%

### Determination of the PAH content

Ten-gram plant sample was grinded and extracted with 20 mL n-hexane. Ten-milliliter acetone was added prior to extraction, and 100 μL of 0.01 μg/mL deuterated PAH surrogate mixture (naphtalene-d8, acenaphthene-d10, phenanthrene-d10, chryzene-d12, benzo(a)pyrene-d12, and perylene-d12, from Restek Corporation, Bellefonte, Pennsylvania, USA) were spiked to it. Dry nitrogen stream was used to concentrate the extract to 1 mL, and an additional solid phase silica gel and alumina oxide sample clean-up was performed. 100 μL of 0.01 μg/mL internal standard (p-terphenyl-d14, 2-fluorobiphenyl from Restek Corporation, Bellefonte, Pennsylvania, USA) was added to the clean sample. The plant samples were analyzed by an HP-6890 gas chromatograph coupled to an HP-5973 (Agilent Technologies, Palo-Alto, USA) quadrupole mass spectrometer (low-resolution single MS). The standards were properly diluted with GC grade solvents (Sigma-Aldrich, St. Louis, Missouri USA) and prepared freshly before the analysis.

During the analysis the head pressure of the column was 50PSI. ZB-Semivolatiles (Phenomenex, Torrance California USA) GC column was used). For 3 minutes after injection, the temperature of the GC oven was 40 °C and heat up (40 °C/min) to 80 °C for 0.5 min.With small breaks, the temperature was increased (15 °C/min) to 310 °C. With constant flow rate (1.2 mL/min) of the helium (N55, Linde Dublin Ireland), carrier gas was used. During the callibration for each target, compound from the standard mixture was estabilished. Five different concentrations between 0.5 and 5.0 ng/mL was detected to determinate the calibration curve.

The measurement was based on MSZ EN 15527:2009 (characterization of waste, determination of polycyclic aromatic hydrocarbons (PAH) in waste using gas chromatography mass spectrometry) (more detailed description was given in Hubai et al. (2021)). The limit of PAH detection (LOD) in extract was 0.001 μg/L and in plant samples 0.1 μg/kg dry plant material.

Analytical determinations were performed by courtesy of the Laboratory of the ELGOSCAR-2000 Environmental Technology and Water Management Ltd. accredited by the National Accreditation Authority, registration number NAH-1-1278/2015.

## Results and discussion

### Measured PAH concentrations in the selected households

**Fig. 1:**
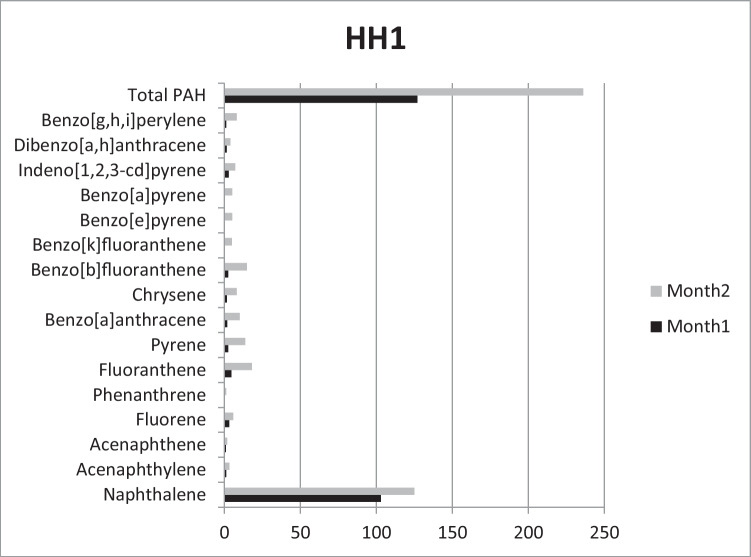
Accumulated PAHs (μg/kg) in Household1 after the first and second months

Prior to the test, PAH determination was carried out. No PAHs were detected. In HH1, concentration of total PAHs showed a clear time dependency, being 127 μg/kg at the end of the first month of the exposure and 236 μg/kg at the end of the second month. LMW PAHs showed this accumulation pattern, e.g., concentration of Acy was 1.2 μg/kg and 3.3 μg/kg, Ace was 1 μg/kg and 1.7 μg/kg, and Flu 3.2 μg/kg and 5.7 μg/kg. On the other hand, some HMW PAHs such as B(g,h,i,)p showed a marked increase in the second month of the exposure, as its concentration was 1.1 μg/kg at the end of the first month but 8.1 μg/kg at the end of the second month. Concentration of the carcinogenic B(a)p was 0 μg/kg at the first half of the exposure period but grew to 5.1 μg/kg by the end of the exposure (Fig. 1.).

In all households, the dominant PAH was Nap (HH1: 103 μg/kg and 125 μg/kg, HH2: 44 μg/kg and 260 μg/kg, and HH3: 195 μg/kg, respectively), in concordance with other studies (e.g., Zhu and Wang 2003, Sharma and Jain 2020).

In HH2, concentration of total PAHs was 133.6 μg/kg and more than tripled by the end of the second month, reaching as much as 411.5 μg/kg. The greatest differences in comparison to HH1 were the lack of certain PAHs: for example, the carcinogenic B(a)p could not be detected. No 6-ring PAHs were detected, either.

**Fig. 2:**
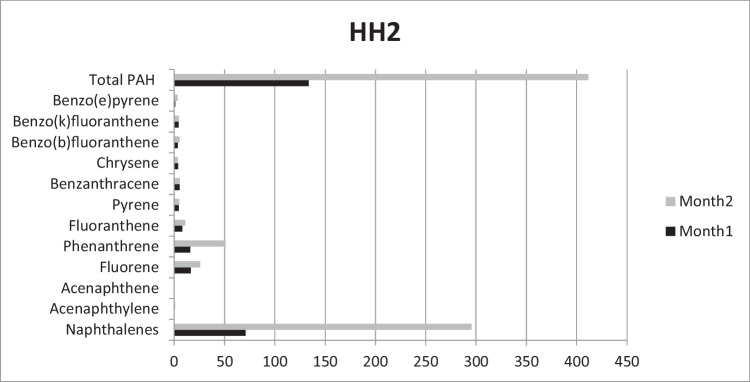
Accumulated PAHs (μg/kg) in Household2 after the first and second months

In HH2, the dramatic increase between Month1 and Month 2 can be attributed to Nap: its concentration was 44 and 260 μg/kg, respectively. Concentration of 3-ring PAHs also showed a marked increase, in case of Phen for example this increase was from 16 to 51 μg/kg. However, concentration of higher MW PAHs showed a less clear tendency: for example concentration of the 5-ring B(e)p increased from 1 to 3.3 μg/kg while concentration of B(k)f remained practically the same (4.3 and 4.4 μg/kg) (Fig. 2).

**Fig. 3:**
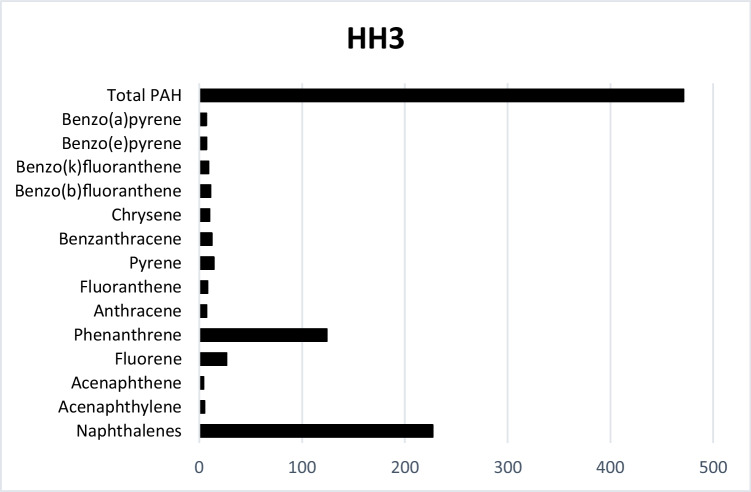
Accumulated PAHs (μg/kg) in Household3 after the first month

In HH3, results are given only for the first month’s exposure, due to the damage of the test system (Fig. 3). Total amount of PAHs, however, reached as much as 471.18 μg/kg. The second dominant PAH was Phen (124 μg/kg). Concentration of the carcinogenic B(a)p was also relatively high, 6.7 μg/kg.

**Fig. 4:**
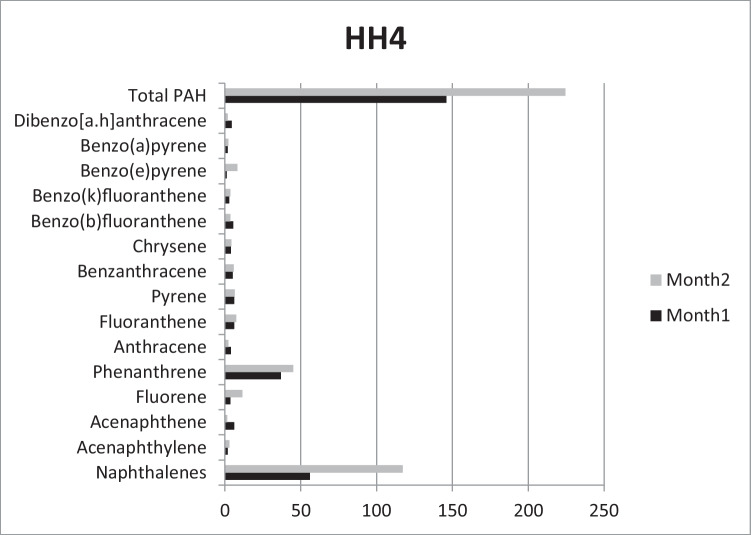
Accumulated PAHs (μg/kg) in Household4 after the first and second months

In HH4 (Fig. 4), total amount of accumulated PAHs was lower than in the other sampling sites, 146 and 224.5 μg/kg by the end of the two exposure periods. Similarly to HH3, the second dominant PAH was Phen (37 and 45 μg/kg, respectively). Concentrations of the carcinogenic B(a)p were rather similar comparing Month1 and Month2, 1.9 and 2.3 μg/kg, respectively. Interestingly, concentration of B(e)p increased from 1.1 to 8.3 μg/kg by the end of the study.

Phen was found at high concentrations in the study of Sun et al. (2020), when different Chinese cooking styles were compared. Phen was characteristic of Sichuan cuisine, which is mixed with quick frying, high-temperature cooking, and large oil consumption. In residential Chinese kitchens, Phen was one of the most characteristic PAHs in the emissions from water-based cooking activities (Zhao et al. 2019). In general, the relatively high share of Phen in HH2–HH4 might raise some human health concern as in the study of Shin et al. (2013) where the main exposure pathway to Nap and Phen was inhalation from indoor sources in developed countries.

See et al. (2006) reported that in general, frying operations such as stir- and deep-frying generated higher molecular weight compounds. These operations are very characteristic of Asian cooking: extremely high amount of IP was reported by Singh et al. (2016) in samples taken in a North Indian commercial kitchen. Zhang et al. (2017) also detected high concentrations of B(a)P, B(b)F, B(a)A, IP, and Cry during domestic Chinese cooking. It might explain the relatively high share of these PAHs in HH1, as deep-frying accounted for approximately 30% of cooking operations in this kitchen. Li et al. (2018) report that in general, cooking oil fumes contain such harmful PAHs as B(a)P, B(b)F, B(a)A, and DBA. The carcinogenic B(a)P was found a significant fraction of particle-bound PAHs in different oils in the study of Chiang et al. (2022) when deep-frying emissions were analyzed. Lin et al. (2022) suggest that cooking oil is the major source of PAH emissions instead of the food being cooked.

HMW PAHs were also found in university canteens and a charcoal-grilled chicken restaurant in Portugal (Vicente et al. 2021). Alves et al. (2021) compared emission of particulate matter (PM_10_, PM_2.5_, and PM_1_) and total volatile organic compounds (TVOCs) during cooking different typical Latin meals such as stuffed chicken, fried mackerel, and fried and grilled pork. In general, emissions from grilled pork contained PAHs in the highest concentration, including HMW PAHs such as IP, D(a,h)a, and B(g,h,i,)p.

Hu et al. (2021) studied the behaviour of PAHs during deep frying with special regard to the mutagenic B(a)P and found that PAH level in sunflower oil generally accumulated with increasing frying time. It might explain the drastic increase of this PAH in HH1, too. In general, B(a)P emission was found characteristic during deep frying in the study of Yao et al. (2015). In our study, it was detected in households using this cooking method such as HH1, HH3, and HH4.

HH1 was also the only household where olive oil was used at relatively high frequency (approximately 5%). Wang et al. (2018) compared particle emission characteristics originated from using four types of oil (soybean oil, olive oil, peanut oil, and lard) and found olive oil emitting the highest number of particles.

### Accumulation capacity of Chlorophytum comosum

Several studies have reported the relatively higher accumulation rate of LMW PAHs (Jia et al. 2018, Wang et al. 2017). Exposure pathways do differ to some extent: more volatile LMW PAHs are available in gas phase, while HMW PAHs are less volatile and occur mostly in particulates (Mukhopadhyay et al. 2020).

On the other hand, accumulation pattern might highly depend on the taxon used (Huang et al. 2018). Capozzi et al. (2017) found that *Robinia pseudacacia* leaves were able to accumulate both LMW and HMW PAHs in a field study. Similar bioaccumulation capacity was reported for the perennial *Plantago lanceolata* (Bakker et al. 1999, Hubai et al. 2021). Relatively high share of 5-ring and 6-ring PAHs was found in Poaceae species such as rice (Tao et al. 2006) and grass (Borgulat and Staszewski 2018). Positive correlation was found between atmospheric and accumulated PAH concentrations in maize (Lin et al. 2007).

**Fig. 5:**
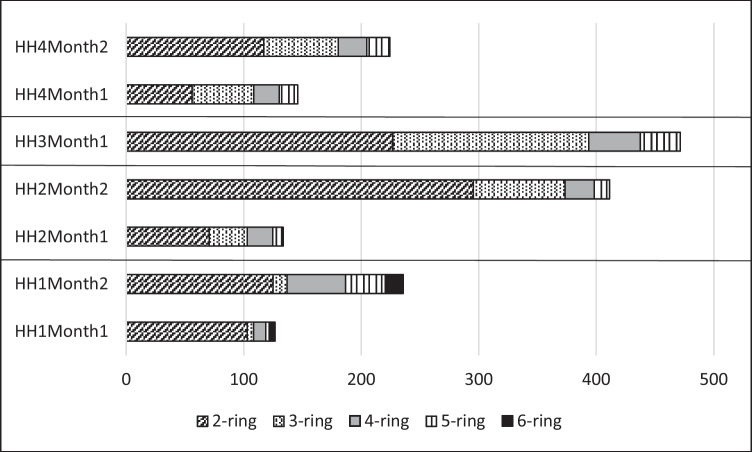
PAH isomers in *Chlorophytum* samples in HH1-HH4

Figure 5 shows the amount of PAH isomers in the different *Chlorophytum* samples. The relatively high share of 5-ring and 6-ring PAHs in HH1 at the end of the total exposure (33.9 μg/kg and 15.1 μg/kg) clearly shows that this species is able to accumulate HMW PAHs. Five-ring PAHs also reach considerable concentrations in *Chlorophytum* leaves in HH2 and HH4 (13 μg/kg and 19.8 μg/kg by the end of the second month, respectively) and 33.6 in HH3 by the end of the first month of exposure.

The formation of HMW PAHs is generally associated with high-temperature cooking such as deep-frying which is done at 170–220 °C (Li et al. 2022). Zhang et al. (2009) reported that these PAHs might also be associated with frying in sunflower oil. Of HMW PAHs, IP was found to be a potential marker for deep-frying which had a high share in HH1 (Xu et al. 2020).

## Conclusions

Accumulation of PAHs was assessed in 4 Hungarian kitchens over 1- and 2-month-long exposures. The selected kitchens shared important characteristic features such as size of the household, but significantly differed considering materials used and cooking practices. Most important difference was the use of sunflower oil and deep-frying as a cooking method. In these households, the relative share of HMW PAHs was considerably higher than in the household which used lard and butter. Deep-frying was also indicated by detectable amounts of the carcinogenic B(a)p. Nevertheless, the dominant PAH was Nap, in concordance with reported studies. PAH accumulation showed a clear time-dependent pattern, as concentrations were considerably higher at the end of the total exposure period (2 months) than after 1-month exposure. *C. comosum* was able to accumulate both LMW and HMW PAHs, so it has proven a sensitive biomonitor for indoor PAH levels. Its use as biomonitor is further increased by the popularity and easy-to-cultivate nature of this ornamental plant.

## Abbreviations

PM: Particulate matter
PAH: Polycyclic aromatic hydrocarbon
LMW: Low molecular weight
HMW: High molecular weight
Nap: Naphthalene
Acy: Acenaphthylene
Ace: Acenaphthene
Flu: Fluorene
Phen: Phenanthrene
Ant: Anthracene
Flt: Fluoranthene
Pyr: Pyrene
B(a)a: Benzo[a]anthracene
Cry: Chrysene
B(b)f: Benzo[b]fluoranthene
B(k)f: Benzo[k]fluoranthene
B(e)p: Benzo[e]pyrene
B(a)p: Benzo[a]pyrene
IP: Indeno[1,2,3-cd]pyrene
D(a,h)a: Dibenzo[a,h]anthracene
B(g,h,i)p: Benzo[g,h,i]perylene
